# Dynamic Changes in Physicochemical Properties and Microbial Diversity During the Fermentation of Mao-Tofu

**DOI:** 10.3390/foods14050775

**Published:** 2025-02-24

**Authors:** Dongqi Li, Yaqiong Wan, Xiaohan Zhou, Juanjuan Cheng, Jieping Zhang, Jianghua Cheng, Yayuan Xu

**Affiliations:** 1 Institute of Agro-Products Processing, Anhui Academy of Agricultural Sciences, Hefei 230001, China; 15345602279@163.com (D.L.); wanyaq@aaas.org.cn (Y.W.); 2 Anhui Engineering Laboratory of Food Microbial Fermentation and Functional Application, Hefei 230001, China; 3 School of Life Sciences, Anhui Agricultural University, Hefei 230036, China; zhouxh1210@163.com (X.Z.); cjj191229@163.com (J.C.); zjp6193600@163.com (J.Z.)

**Keywords:** Mao-tofu, microbial community, biogenic amines, free amino acids

## Abstract

Mao-tofu is famous for its unique flavour and texture in Anhui Province, China. The physicochemical properties and microbial diversity of Mao-tofu during different fermentation periods were studied. The pH of the tofu was acidic, the moisture gradually decreased, and the hardness, viscosity, and chewiness gradually increased, while the elasticity gradually decreased. Among these, changes in volatiles and synergistic effects of proteins, peptides, and free amino acids (FAAs) affect the flavour of Mao-tofu. Histamine had the highest concentration among all biogenic amine (BA) during the whole fermentation process. A microbial community analysis showed that *Lactobacillus* and *Trichosporon* were the most important strains throughout the fermentation process. Bacterial abundance and diversity also showed a gradual increase, while fungal abundance and diversity showed a gradual decrease. A comprehensive analysis of the physicochemical changes associated with microbial succession can help to gain insights into the maturation process of texture and flavour during the production of Mao-tofu.

## 1. Introduction

Mao-tofu is a traditional folk food with a unique flavour in China that has thousands of years of history [1]. Through the steps of pulping, pulping, moulding, and cutting the and emulsification of soybeans, artificial fermentation causes a layer of white fuzz to grow on the surface of the tofu. According to legend, after Zhu Yuanzhang captured Huizhou in the 17th year of the Yuan Dynasty (1357), the people in the area treated their soldiers with watery tofu. The watery tofu took more than a moment to eat, and the heat made the tofu grow white and brown fuzz. In order to prevent waste, Zhu Yuanzhang ordered the cook to deep fry and then stew the tofu with a variety of seasonings, which produced the distinctive flavour of the Mao-tofu. It is rich in nutrition, possesses a unique flavour, and offers a fresh taste. Free amino acids (FAAs), such as glutamic acid and aspartic acid, and some peptides and nucleotides play an important role in the umami flavour of Mao-tofu. The fermentation process of Mao-tofu entails the dynamic succession of microbial communities. In this process, these microorganisms will produce various enzymes to make Mao-tofu more easily absorbed by the body [2]. The fermentation process of Mao-tofu benefits from the abundance of microorganisms, which contribute to a rich enzyme system, including protease, cellulase, pectinase, and β-glucosidase. These enzymes play a crucial role in decomposing the protein, starch, and lipids in tofu into small-molecule flavour substances and nutrients enzymatically. Consequently, this enzymatic action softens the tofu’s texture while enhancing its overall flavour profile. Fungi are particularly important in Mao-tofu fermentation because they contribute to pronounced fluffiness. It is assumed that the main fungi of Mao-tofu are mucors [3,4,5].

Bioamines are nitrogenous organic compounds commonly detected in protein-rich fermented foods. Bioamine production in fermented soy products has been attributed to the action of multiple decarboxylases that promote FAA decarboxylation [6,7], which are converted to bioamines by microbial metabolism. Low bioamine levels may be important in promoting cell growth and division, but an excessive intake of bioamines from food can cause physiological symptoms such as headaches, nausea, breathing difficulties, heart palpitations, and high blood pressure. Lactic acid bacteria in fermented foods result in biogenic amine (BA) production, and multiple genes associated with the BA pathway have been identified in *Lactobacillus* [8,9,10].

However, despite its extensive history of production and consumption, little is known about microbial community dynamics, physicochemical property variations, and BA content alterations during fermentation and maturation processes. Furthermore, the potential role and relationship between microbial communities and these physicochemical properties have yet to be fully elucidated. Therefore, it is necessary to further investigate the diversity and microbial structure of Mao-tofu. This study aimed to investigate the succession and physicochemical changes of bacterial and fungal communities, assess the interrelationships between microbiota and physicochemical factors, and enhance our understanding of the taste and flavour maturation process of Mao-tofu during maturation.

## 2. Materials and Methods

### 2.1. Sample Collection

**Figure 1:**
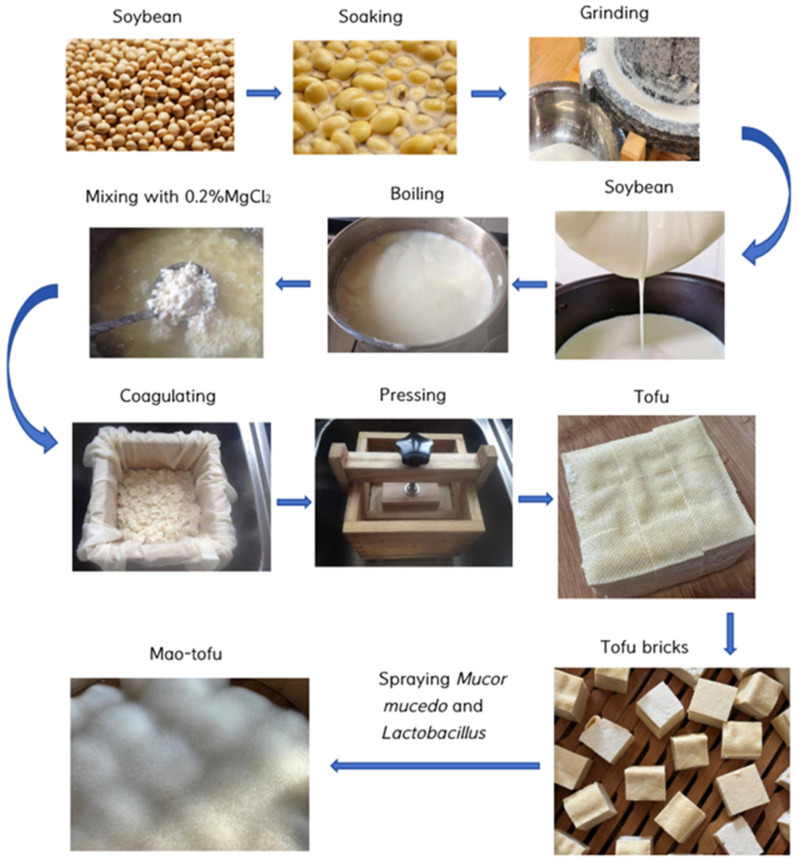
Traditional manufacturing process of Mao-tofu.

In order for the dal to expand sufficiently to facilitate grinding, the soybeans (the Anhui Hefei Zhonghuang 901 variety of raw soybean materials was purchased from the Anhui Hefei Zhongcai market, for a total of one batch) were thoroughly cleaned and soaked at room temperature (25 °C) for 12 h with an appropriate amount of water. Six times the amount of water was added to the hydrated soybeans, and the solution was mixed, ground, and filtered to obtain soybean milk. Pure water (distilled water) was stirred continuously at 95 °C for 10 min before cooling to 80 °C. A 0.2% MgCl_2_ solution was added to the hot soybean milk, stirred for 5 min, and allowed to stand for 15 min to solidify. The solidified tofu was pressed into thin slices and cut into small pieces measuring approximately ~2 cm × 2 cm × 2 cm. The laboratory-inoculated tofu was treated with pure cultures of *Mucor mucedo* and *Lactobacillus paracasei* (CCTCC patent nos. M20211074 and M20211073; National Center for Biotechnology Information: MW080631.1 and MW080365.1, respectively), both of which were high-yielding protease strains screened from coarse tofu in the Huizhou area and incubated at 20 °C for 3 days. After 3 days of cultivation, the mycelium-covered hairy bean curd was obtained. The samples were collected aseptically in disposable sterile boxes at 0, 12, 24, 36, 48, 60, and 72 h fermentation. Three replicates were taken at each time point and stored at 4 °C (Figure 1) [11].

### 2.2. High-Throughput Sequencing (HTS)

Next-generation sequencing (including sample DNA preparation, polymerase chain reaction [PCR] amplification, and Illumina MiSeq sequencing and analysis) of Mao-tofu was conducted at different fermentation times [12].

Sample DNA preparation: The genomic DNA of Mao-tofu samples was extracted using the sodium dodecyl sulphate method, and DNA purity and concentration were detected using agarose gel electrophoresis. The sample DNA was placed in a centrifuge tube and diluted with sterile water, and the experiment group was tested in parallel at least thrice.

PCR amplification: The V3 and V4 regions of the 16S rRNA were amplified using the universal 16S primer to investigate the presence of standard bacteria, with the diluted genomic DNA of Mao-tofu samples serving as the template. Similarly, ITS1 rDNA was amplified with a universal primer to examine the occurrence of standard fungi.

Illumina MiSeq sequencing and analysis: Amplicon libraries were prepared and sequenced on a MiSeq Illumina platform with version 3 300 PE chemistry.

### 2.3. BA Determination

BA determination was conducted by high-performance liquid chromatography (HPLC)-tandem mass spectrometry (MS) using the methods described by Gu et al. [13].

HPLC (LC2023C3D; Shimadzu, Kyoto, Japan) and a Waters C18 column (2.1 mm × 100 mm, 1.7 μm) were used. The mobile phase A was set at a 0.1% acetonitrile format, and the mobile phase B was set at a 0.5% formic acid aqueous solution. Column temperature: 35 °C; sample size: 2 μL. The gradient elution procedure is shown in Table 1.

For HPLC calibration, the standards for humetine, cadherine, tyramine, tryptamine, B-phenethylamine, histamine, spermidine, 1,7-diaminoheptane (internal standard), and benzyl chloride were purchased from Chunqiu Bioengineering Co., Ltd. (Nanjing, China). The peak area internal standard method was used for quantitative determination, and the BA content was expressed in mg/L. All experiments were repeated thrice.

### 2.4. Physiochemical Analysis

A TA.XTplusC Texture Analyser (Stable Micro Systems Products, Godalming, UK) was used to test the hardness, viscosity, chewiness, and elasticity of the Mao-tofu. The Mao-tofu was cut into cubes measuring 2 × 2 × 2 cm^3^ and compressed for 3 s at a constant speed of 2 mm/s using a stainless-steel plunger with a diameter of 35 mm. A trigger force of 5 g was applied, resulting in a deformation of 45%. The water content of the Mao-tofu was analysed by the direct drying method; the sample was placed in the 101~105 °C drying oven for 4 h and then covered and removed, put into the desiccator to cool down for 0.5 h, then weighed, then put into the 101~105 °C drying oven for 1 h or so, removed, and put into the cooled desiccator for 0.5 h, and then weighed. Its pH was determined by a pH meter [14,15]. The content of individual FAA was determined by an amino acid analyser using post-column derivatization. The separation was performed on a cationic resin exchange column with a detection wavelength of 570,440 nm and a column temperature of 58–74 °C. The flow rate of the elution pump was 0.45 mL/min, and the flow rate of the derivatization pump was 0.25 mL/min with the derivatization reagent ninhydrin [16]. A headspace solid-phase microextraction (HS-SPME) (HP-5MS flexible quartz capillary column [30 m × 250 μm, 0.25 μm]) of volatile compounds was performed, and the extraction results were analysed by a gas chromatography–mass spectrometry (GC–MS) system (8890/7000D GC-MS coupler) [17]; the protein and peptide contents in the Mao-tofu were quantified by the trichloroacetic acid soluble nitrogen method (TCA-NSI) [18].

### 2.5. Statistical Analysis

All tests were performed thrice. A statistical analysis was performed using IBM SPSS Statistics 26, with a significance level set at *p* < 0.05. The HTS results adopted operational taxonomic units (OTUs) [19] to study the diversity of the microbiota. The main flavour components of the Mao-tofu were determined by the gas chromatography (GC)–MS technique and comparison MS library. The data were plotted using Origin 2023 and GraphPad Prism 8.0.2. Canoco 5 was used for the redundancy analysis (RDA), and Excel was used for the table drawing.

## 3. Results and Discussion

### 3.1. OTU Distribution in Mao-Tofu and Microbial Species

**Figure 2:**
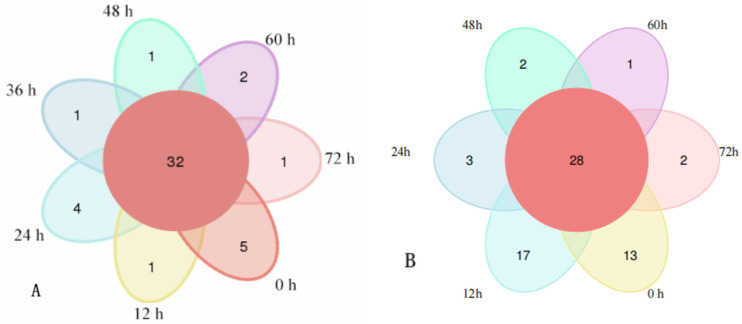
Venn diagram of OTU distribution of bacteria (**A**) and fungi (**B**) in fermented Mao-tofu samples.

Venn spots were constructed from the HTS detection results. Figure 2 shows the unique and common OTU numbers in the Mao-tofu samples and the similarity and overlap of their composition. In Figure 2A, there were 32 overlapping bacterial OTUs in the Mao-tofu samples at 0, 12, 24, 36, 48, 60, and 72 h, accounting for 68.08% of the total OTUs. That is, the fermentation of the Mao-tofu samples during this period was unique. The numbers of OTUs in this period were 5, 1, 4, 1, 1, 2, and 1, respectively. The difference between bacteria and microorganisms in the samples was small. In Figure 2B, there were 28 overlapping fungal OTUs, accounting for ~42.42% of the total OTUs. This indicated some differences in fungal microorganisms during fermentation at 0, 12, 24, 48, 60, and 72 h, suggesting that the flavour compounds in the Mao-tofu may be determined by unique microbiota.

### 3.2. Microbial Species Diversity Curves in Mao-Tofu

The diversity of different sequenced microorganisms and the sequencing amount of different Mao-tofu samples were also different. The dilution and rank–abundance curves were obtained by HTS detection [20]. The dilution curve was established using the Mao-tofu microbial diversity as the index, and it exhibited a positive correlation with the sample size. Furthermore, the establishment of the dilution curve was based on the assessment of the Mao-tofu microbial diversity, whereas an increase in the sample size resulted in a flatter dilution curve. The grade abundance curve reflected the phase richness and evenness of the microflora, with its shape as an indicator of uniformity in microbial species composition across different fermentation stages. The richness of species was reflected by the length of the curve on the horizontal axis. The wider the line, the richer the species. The uniformity of the species composition was reversed by the shape of the curve. The flatter the curve, the more uniform the species composition.

**Figure 3:**
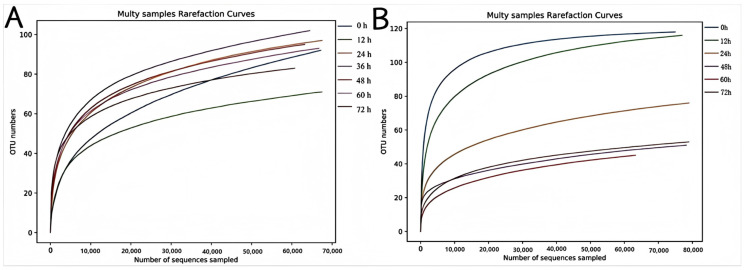
Bacterial (**A**) and fungal (**B**) dilution curves.

The dilution curves of the bacteria (Figure 3A) and fungi (Figure 3B) can fully reflect the diversity of the microbial flora. Figure 3A demonstrates that the coverage index of the sample exceeded 99.9%. Although the refraction curve did not reach saturation, the number of newly detected species declined as sequencing depth increased; ultimately, the curve levelled off. The sequencing depth of the samples met the requirements, and the main types of microorganisms in the Mao-tofu could be detected. The length of the transverse axis of the curve reflects the species richness. The species richness and diversity of the bacterial community in the samples changed throughout the fermentation process. The richness and diversity of the bacterial community in the samples were significantly lower than those in other samples at 12 h fermentation, suggesting fewer bacterial species in fresh billets. As the Mao-tofu fermentation progressed, the richness and diversity of the bacterial community increased gradually, and the diversity reached the maximum value at 48 h fermentation. This indicated that microorganisms in the environment were enriched in the Mao-tofu, and the bacteria present in the Mao-tofu began to adapt to the environment and efficiently utilize nutrients from raw materials for rapid growth and multiplication. Subsequently, the bacterial diversity exhibited a decrease at 60 h. This could be attributed to the fact that microorganisms grow and multiply rapidly during the preproduction period of Mao-tofu, resulting in a significant number of secondary metabolites, especially acids, which inhibit acid-sensitive bacterial growth. In Figure 3B, the diversity and richness of the fungal community gradually decreased with the increase in fermentation time, and the bacterial richness was lowest at 60 h and increased at 72 h. It was speculated that some fungi began to grow in the hairy tofu after 60 h fermentation, and the richness of the fungal community increased slightly. The specific reasons need further analysis.

**Figure 4:**
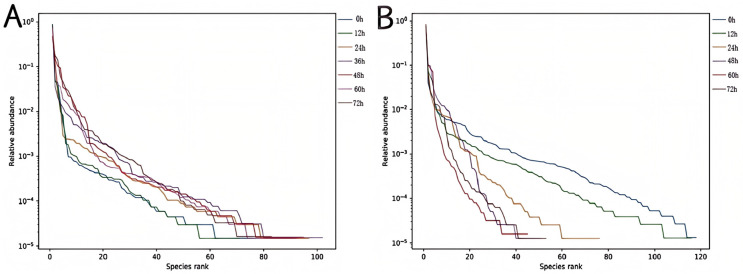
Bacterial (**A**) and fungal (**B**) rank–abundance curves.

In Figure 4A, the transverse axis of the curve was widest at 36 h, narrowest at 0 h, and second widest at 12 h. The results showed that the bacterial composition of the Mao-tofu was higher than that of other groups after 36 h fermentation, and the bacterial species of the Mao-tofu were more abundant. The curves of different fermentation periods were relatively flat, indicating that bacterial community composition was relatively uniform. In Figure 4B, the transverse axis of the curve was widest at 0 h and narrowest at 60 h. The results showed that the fungal composition of the fermented Mao-tofu was lower than that of the unfermented fresh tofu, indicating that with the extension of fermentation time, the fungal abundance decreased, and the diversity and variability of the fungal species decreased. The longer fermentation time, uneven curve, and different fermentation times on the surface of the Mao-tofu microbial diversity were obviously different.

### 3.3. Analysis of Microbial Species Differences in Mao-Tofu

In this experiment, we used the high protease- and aminopeptidase-producing strains of *Mucor mucedo* and *Lactobacillus paracasei*, which were screened by the laboratory from fermented soybean products in the northern Anhui Province, as inoculants for the mixed fermentation of the Mao-tofu, which produces a large number of proteases and aminopeptidases in the process of fermentation to enzymatically dissolve the large-molecule proteins into small molecules of free amino acids, which are more readily absorbed, and which has a distinctive flavour.

**Figure 5:**
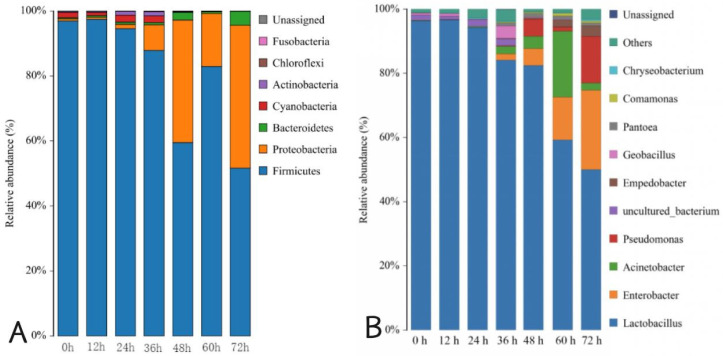
Distribution of bacterial community structures in fermented Mao-tofu samples at the phylum (**A**) and genus (**B**) levels.

The microbial community succession of the Mao-tofu was studied in different fermentation stages through phylum- and genus-level classification. In Figure 5A, Firmicutes and Proteobacteria were the predominant phyla in fermentation, and their relative abundances were 96.97% and 0.64%, respectively, at 0 h fermentation. At 12 h fermentation, these proportions slightly increased to 97.47% and decreased to 0.59%. After 24, 36, 48, 60, and 72 h fermentation, the relative abundance ratio of Firmicutes was 94.61%, 87.88%, 59.49%, 82.94%, and 51.64%, respectively. Similarly, the relative abundance of Proteobacteria after fermentation for the same time intervals was 1.31%, 7.89%, 37.73%, 16.30%, and 43.99%, respectively. Firmicutes accounted for 97.47% at 12 h, representing the highest proportion (*p* < 0.05).

The bacterial community in the fermentation process of the Mao-tofu consisted of 154 genera of microorganisms. Figure 5B shows that the bacterial community is mainly composed of nine genera (relative abundance > 0.5%): *Lactobacillus*, *Enterobacter*, *Acinetobacter*, *Pseudomonas*, *Empedobacter*, *Geobacillus*, *Pantoea*, *Comamonas*, and *Chryseobacterium*. The *Lactobacillus* genus held a dominant position in the fermentation process, serving as the primary bacteria responsible for the dominant microbial during fermentation. *Enterobacter*, *Acinetobacter*, and *Pseudomonas* were dominant at 48 h fermentation. The abundance of *Enterobacter* spp. gradually increased with the fermentation time after 36 h fermentation. The abundance of *Fusobacterium* spp. increased and decreased after 36 h, reaching its peak at 60 h fermentation. The abundance of *Pseudomonas* spp. exhibited an initial increase, followed by a subsequent decrease during the middle and late fermentation stages (36–72 h). Subsequently, it displayed a renewed increase with undulating changes in the band, demonstrating a negative correlation with the changes observed in *Fusobacterium* spp. *Geobacillus* spp. functioned mainly in the prefermentation period, decreased rapidly as fermentation progressed, and remained low (relative abundance < 1.0%) in the late fermentation period. The other bacterial species, *Empedobacter*, *Pantoea*, *Comamonas*, and *Chryseobacterium*, mainly played a role in the late fermentation stage and gradually increased with the process. In addition to the nine major bacteria, at the beginning of fermentation (0 h), uncultured and unassigned bacteria were the main microbial groups, but their relative abundance decreased significantly with fermentation and remained below 1.0% after 48 h. This suggested that the fermentation environment of Mao-tofu is unsuitable for developing microorganisms because of the selective pressure on exogenous microorganisms. Because Mao-tofu is made from fermented “acidic syrup water”, the pH value is acidic, and the growth of many bacteria is inhibited, with *Lactobacillus* spp. as the dominant strain. The simulated coagulation conducted by Yan confirmed that *Lactobacillus ultunensis* is associated with superior tofu quality. This finding suggested that *Lactobacillus delleri* may play a core role in flavour coagulation and production [21]. *Lactobacillus* is a common probiotic in fermented foods, which can produce various chemical substances such as organic acids, aromatic compounds, and extracellular polysaccharides during the fermentation process, and can also provide the whole lactobacillus system with various bioactive substances such as organic acids, diacetyl, hydrogen peroxide, and bacteriocins, which not only effectively improves the organoleptic quality, but also inhibits the formation of undesirable microorganisms [22]. *Lactobacillus* can secrete proteases and aminopeptidases, which have the ability to decompose protein [23]. *Bacillus* plays a crucial role in soybean fermentation by secreting microbial inhibitors and fibrinogen, which can enhance the inhibitory effect of soybeans and its anthocyanins on angiotensin-converting enzyme content and reducing activity [24]. *Bacillus* also enhances lipases, amylases, proteases, cellulases and glutaminases, which degrade lipids, proteins, carbohydrates, and flavonoid glycosides during fermentation to produce metabolites such as organic acids, amino acids, and glycosides, which contribute to flavour, taste, functionality, and nutritional composition [25]. In the preliminary study of bacteria in the tofu by pure culture, the bacterial community in the tofu was mainly composed of *Lactobacillus* spp., *Enterobacter*, and *Bacillus*. This was essentially the same as the obtained results. Another noteworthy point is that the relative abundance of unclassified bacteria throughout fermentation was 1.3%, which is related to the available reference sequence information. With the improvement of the annotation information, it will be beneficial to our understanding of currently unknown bacteria.

**Figure 6:**
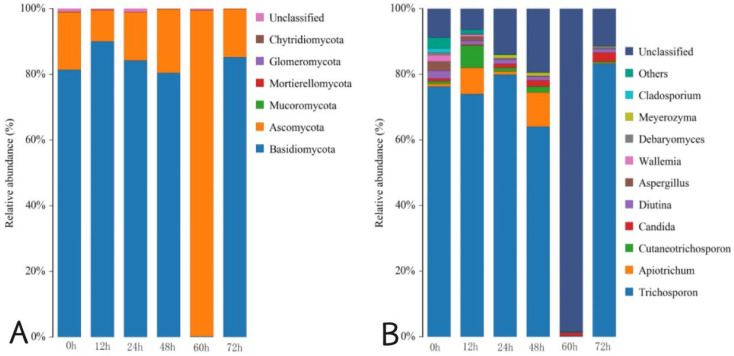
Distribution of fungal community structures in fermented Mao-tofu samples at the phylum (**A**) and genus (**B**) levels.

In Figure 6A, at the phylum level, there were six phyla in Mao-tofu fermentation: Basidiomycota, Ascomycota, Mucoromycota, Mortierellomycota, Glomeromycota, and Chytridiomycota. The average relative abundance of Ascomycota at 0 h fermentation was 17.52%. The mean relative abundances of Basidiomycota at 0, 12, 24, and 48 h were 81.42%, 89.97%, 84.23%, and 80.42%, respectively. At 60 h fermentation, a significant increase in the relative abundance of Ascomycota was noted, reaching up to an expressive value of 99.32%. However, by the completion of the fermentation process (72 h), there was a notable divergence in the average relative abundances between Basidiomycota (85.19%) and Ascomycota (14.72%). Although Ascomycota exhibited dominance with a proportion as high as 99.61% at 60 h, this proportion drastically declined to only 14.72% at 72 h.

Figure 6B shows that at the genus level, there were 10 dominant fungi in the Mao-tofu during fermentation: *Trichosporon*, *Apiotrichum*, *Cutaneotrichosporon*, *Candida*, *Diutina*, *Aspergillus*, *Wallemia*, *Debaryomyces*, *Meyerozyma*, and *Cladosporium*. *Trichosporon* was the dominant fungal group in the fermentation process, and its relative abundance was 76.27% at 0 h fermentation. After 12 h fermentation, *Trichosporon*, *Apiotrichum*, and *Cutaneotrichosporon* were the dominant strains in relative abundance (73.98%, 7.97%, and 6.76%). After 24 h fermentation, *Trichosporon* became a single dominant strain again, and its relative abundance ratio reached 79.85%. After 48 h fermentation, the relative abundances of *Trichosporon* and *Apiotrichum* were 64.03% and 10.37%, respectively. The relative abundance of *Candida* was 0.89% after 60 h fermentation, and it increased by 2.71% after 72 h fermentation. Before 60 h fermentation, the richness of the fungal community decreased with the increase of time, and after 60 h fermentation, the richness of *Candida* also increased to some extent with the increase of relative abundance, consistent with the results in the fungal dilution curve in Figure 3B. The highest proportion of *Trichosporon*, which reached 79.85%, was observed after 12 h during fermentation.

The composition of the fungal microbialin in the Mao-tofu samples was different from that of many other fermented soybean products. For example, *Actinomyces* spp. and *Trichoderma* spp. are the most common microbiota in curd [26]. In contrast, the most dominant fungal taxa in Mao-tofu are yeasts, followed by moulds. The nutrition, flavour, and texture of Mao-tofu are highly related to the presence of fungi during the fermentation process. Moulds can produce precursors of aromatic substances and enzymes such as proteases, peptidases, and amylases, which break down starch and proteins into peptides and amino acids and promote the hydrolysis of nutrients in the food, which are then absorbed and utilised by the yeast [27]; the yeasts convert glucose into glycerol, ethanol, isobutanol, isoamyl alcohol, and other substances that contribute to flavour [28].

### 3.4. Cluster Analysis and Discrepancy Species Analysis

**Figure 7:**
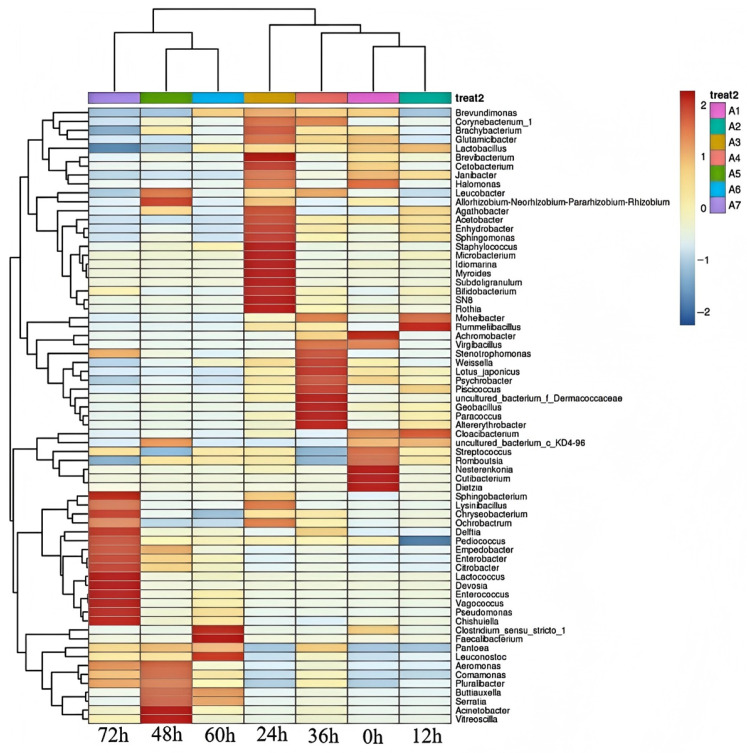
Analysis of the differences between Mao-tofu groups. Cluster analysis of the bacterial community at different fermentation stages.

In Figure 7, a cluster analysis was performed to investigate the dynamic succession of microbials during the fermentation stages. The samples were grouped into three distinct clusters. All samples, including the 48, 60, and 72 h fermentation samples, exhibited a stronger tendency to cluster together within Cluster 1. Cluster 2 comprised all stage samples taken at 0, 12, 24, and 36 h fermentation. These results indicated that the microbial community structure among different fermentation stages was different, whereas the community structure of external and internal microbes was similar overall.

**Figure 8:**
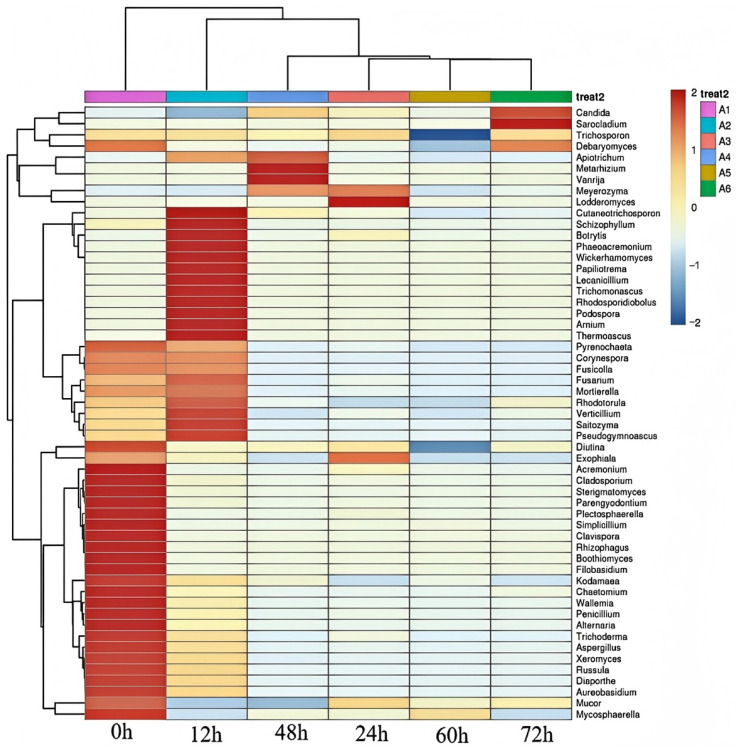
Analysis of differences between Mao-tofu groups. Cluster analysis of the fungal community at different fermentation stages.

In Figure 8, a cluster analysis was performed to study the dynamic succession of fungal microbials during the fermentation stage. All samples fell into one category. The 72 h and 60, 24, and 48 h fermentation samples exhibited higher polymerization levels when combined. Among these four samples, the 24 h fermentation samples showed relatively good polymerization. Consequently, the samples fermented for only 12 h displayed the lowest degree of polymerization. The above results indicated significant differences in the microbial structure of the Mao-tofu depending on their location.

### 3.5. Bacterial Function Prediction

**Figure 9:**
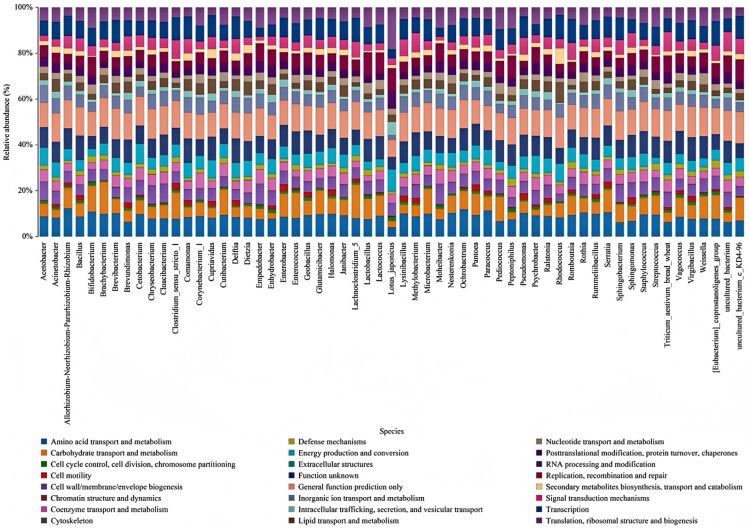
Bacterial function prediction of Mao-tofu.

Based on the sequencing results of 16S rRNA genes, the results from predicting bacterial gene and functional unit composition were summarized. Furthermore, leveraging abundance information at the OTU level, PICRUSt was employed for the prediction of the bacterial functional genes using the Kyoto Encyclopedia of Genes and Genomes (Figure 9). The results showed that bacteria in the Mao-tofu samples were mainly involved in pathways such as cell membrane translocation, signalling protein interactions, amino acid metabolism, carbohydrate metabolism, enzyme synthesis and metabolism, and nucleic acid metabolism. These bacteria, including *Lactobacillus*, *Geobacillus*, *Enterobacter*, *Acinetobacter*, *Pseudomonas*, *Empedobacter*, *Pantoea*, *Comamonas*, and *Chryseobacterium*, are involved in various biochemical reactions and the synthesis and metabolism of polysaccharides and nucleic acids. These bacteria and others can regulate amino acid metabolism and the biosynthesis/metabolism of amines and polyamines. This suggested that certain bacteria in the Mao-tofu samples may contain decarboxylase or amine-degrading enzyme-encoding genes capable of generating BAs via FAA metabolism or degrading BAs through amine-degrading enzymes. Meanwhile, based on the prediction of bacterial biosynthesis and metabolism of amines and polyamines, this study can provide a theoretical basis for the subsequent screening of amine-reducing strains from Mao-tofu.

### 3.6. Changes in Physicochemical Properties During Fermentation of Mao-Tofu

**Figure 10:**
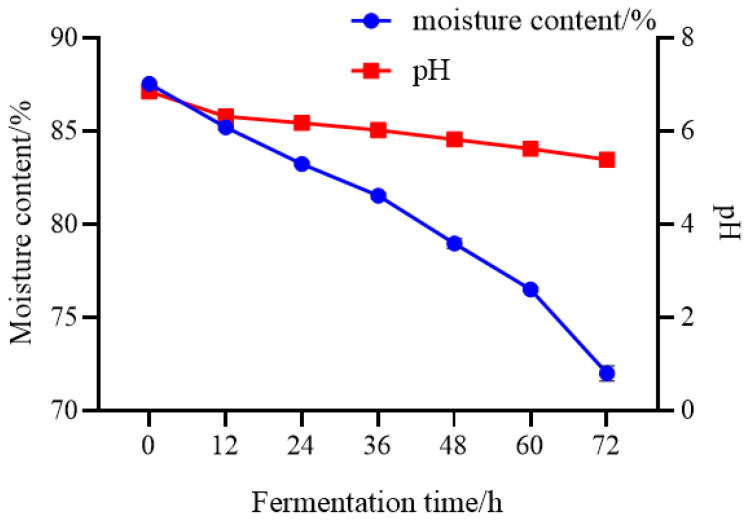
Changes in moisture and pH content during fermentation of Mao-tofu.

The physicochemical properties of the Mao-tofu samples showed significant differences at different stages of fermentation (Figure 10). The samples had moderate levels of pH and moisture at different fermentation times. The decreasing water and decreasing pH with increasing fermentation time was attributed to the fact that during the fermentation process of Mao-tofu, proteins interacted with other proteins or water to form gels, which led to decreasing water during the process [29], and the natural acidification of the yellow slurry water produced during the formation of the tofu further promoted tofu coagulation, i.e., the pH was acidic and decreasing during the fermentation process.

As shown in Table 2 and Table 3, the richest and most important compound in tofu flavour is acid. It affects the consistency of the final product. During the fermentation process of Mao-tofu, microorganisms metabolise and form acids using the sugars in the raw materials. Organic acids not only give Mao-tofu a unique flavour, but also can be converted into esters to further increase the flavour of Mao-tofu. Lactic acid can give Mao-tofu a rounded and elongated flavour [30]. A moderate number of organic acids can prevent the growth of contaminating bacteria and enhance the mellow flavour of Mao-tofu. The samples were rich in organic acids. Linoleic and oleic acids contributed to the fatty odour and green soap consistency of the Mao-tofu; succinic acid has a sour and fresh flavour presentation, and prolongs the shelf-life of the food [31]. Secondly, rich esters were also detected in the samples. The total alcohol content was also found to affect the flavour of the Mao-tofu, with 1-hexanol giving a mung bean flavour and phenylethanol giving a honey rose flavour [32]. A total of five Aldehydes were detected in the samples. Aldehydes, which are formed by the oxidation and breakdown of lipids during fermentation, have a nutty and sweet flavour and can improve flavour [33]. Three Alkenes were present in all tofu samples, but more analysis is needed to determine their relationship to the flavour and aroma of tofu. Two ketones were detected in the samples, which are formed when amino acids or lipids are metabolised by martensitic reactions or fungal enzymes [34]. Two volatile phenols were detected in the samples, which have a spice-like aroma and may be the result of the enzymatic degradation of coumaric and ferulic acids [35]. In contrast, the furans found in the samples were produced by a Meladic reaction during sugar dehydration. With a sweet flavour similar to chocolate and caramel [36], 2-Aminofuran has been reported to have flavour-altering properties in soybean oil. Indole was present in all samples. It is probably produced by the degradation of tryptophan. Indole smells like animal faeces, which gives Mao-tofu its distinctive odour [37].

### 3.7. Textural Changes in Mao-Tofu During the Fermentation Process

Shortly after inoculation with a Mucor starter culture, the fermented Mao-tofu was colonized by fungi, and the mycelia spread on its surface. The physical properties of the Mao-tofu underwent significant changes due to fungal growth and enzymatic degradation. The changes in the physical properties of Mao-tofu during fermentation were investigated (Table 4). The hardness and viscosity of the tofu matrix increased with prolonged fermentation time, whereas the blank decreased, and the texture became harder. Due to the destruction of the inner spatial structure of the tofu under the action of protease, the texture of the Mao-tofu became even and delicate [38]. The elasticity of the tofu substrate decreased with the extension of the fermentation time, and the chewiness of the tofu substrate increased. Overall, the texture of fermented Mao-tofu is better than that of tofu, demonstrating the original organizational state of tofu and offering a better taste.

### 3.8. Changes of Proteins, Peptides, and FAAs During Fermentation of Mao-Tofu

**Figure 11:**
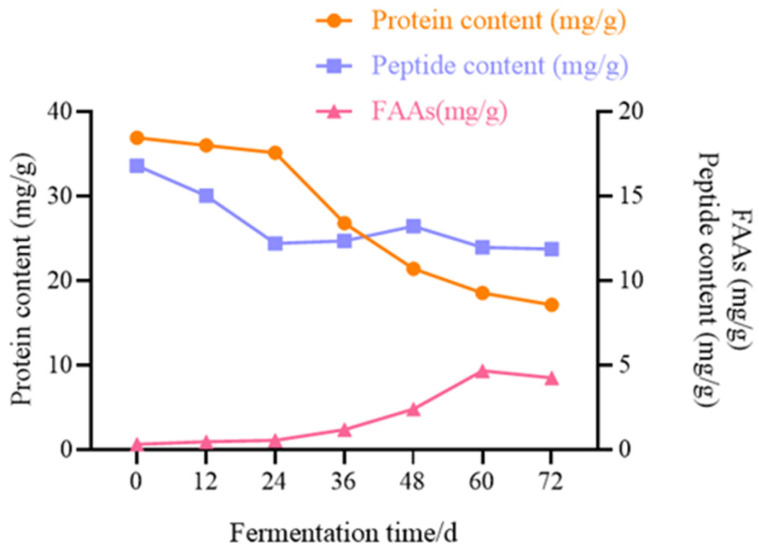
Changes in proteins, peptides, and amino acids in different fermentation times of Mao-tofu.

Fermentation converts structural proteins into amino acids to improve digestibility, and during fermentation, proteins were degraded into peptides and free amino acids, as shown in Figure 11. Protein content decreased with increasing fermentation time, and protein concentration decreased significantly from 35.141 mg/g to 26.81 mg/g after 24 h, whereas crude polypeptide concentration initially decreased and then increased at 24 h before stabilising at 70 h. The total free amino acid concentration peaked at 60 h (4.683 mg/g) and then decreased. In Table 5, the change in total amino acids showed a gradual increasing trend from 0 to 60 h, but at 72 h fermentation, the amino acid content exhibited a sharp decrease. The possible reason for this result may be the increasing amino acid content as fermentation time extends, along with the enhanced hydrolysis of proteins by amino peptidase and protease. However, as the fermentation time increases, the ability of aminopeptidase and protease to hydrolyse proteins and produce amino acids is inhibited, resulting in a lower amino acid content. Conversely, the degradation reaction may be the main reason for reducing the amount of FAA, which is further degraded into other compounds, such as volatile compounds [39]. In contrast to the tofu before fermentation, the total FAA content in the ripe stage of the Mao-tofu increased 14-fold. However, the arginine content exhibited the highest concentration among all FAAs. From the initial period to 60 h, it gradually increased, reaching its peak value of 546.4 mg/kg at 60 h. However, afterward, the content decreased as the fermentation time extended. At the end of fermentation, it accounted for 12.41% of the total amino acids. Energy is required for the growth of microorganisms during fermentation. During the initial stage of environmental adaptation, microorganisms need to accumulate large amounts of nutrients [40]. As a result, most of the proteins, peptides, and small molecule oligopeptides were utilised. This led to the observed decrease in the concentration of proteins and crude polypeptides. Meanwhile, a large number of enzymes such as proteases and aminopeptidases produced by the metabolism of the microbial community during the fermentation process promoted the degradation of proteins in the cytoplasm [41], whereas due to the sustained decrease in environmental pH, the activities of proteases such as endopeptidases and exopeptidases were amplified to further promote protein degradation [42]. As the microorganisms consumed protein nutrients at a slower rate than protein degradation, free amino acid and crude polypeptide concentrations increased steadily after 24 h and peaked at 60 h. The concentration of free amino acids and crude polypeptides increased steadily after 24 h and peaked at 60 h. Although the crude peptide concentration after fermentation was lower than the initial amount, this may be due to the microbial enhancement of enzyme–substrate interactions during the fermentation process, which promotes protein breakdown into smaller peptides and free amino acids [43]. Microbial fermentation produces proteases that can specifically cleave proteins, leading to different bioactive peptides, which are hydrolysed by peptidases to produce tasty amino acids [44]. However, arginine, alanine, and lysine play a major role in the sweetness and bitterness of the food, respectively, suggesting that sweetness and bitterness are the main flavour profiles of Mao-tofu. This study agrees with a previous study that the most abundant amino acids in Mao-tofu are Arg, Ala, Lys, Leu, Phe, and Pro [45]. Therefore, the present study focused on the key microorganisms responsible for the degradation of proteins to crude peptides from 24 to 60 h. The reason for the decrease in the concentration of proteins, natural peptides, and free amino acids after 60 h may be due to the fact that as the microorganisms consume more, there is a lack of carbon or nitrogen sources, and the microorganisms utilise substrate compounds to fuel their growth [46].

**Figure 12:**
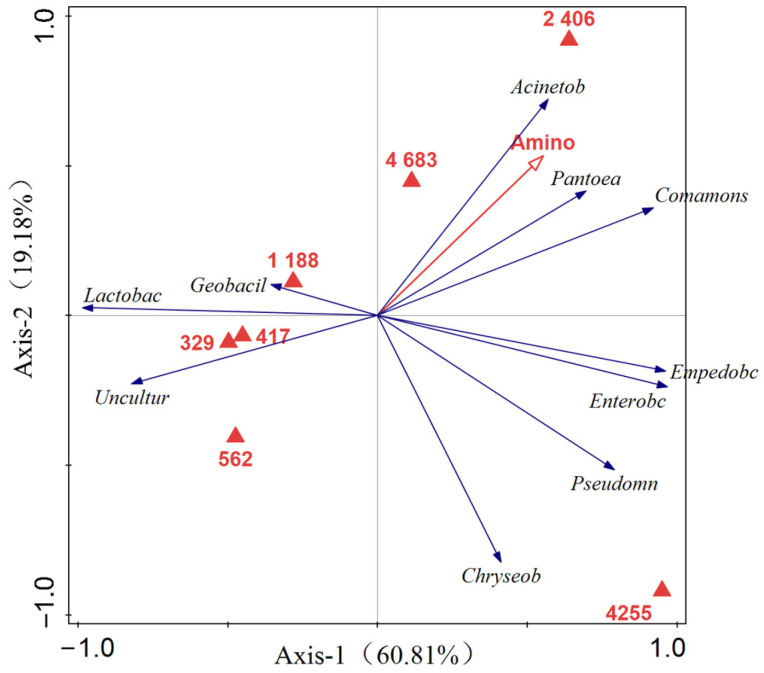
RDA of dominant bacteria and amino acids in Mao-tofu.

The correlation between the top 10 bacterial genera with relative abundance in the samples and environmental variables was obtained through RDA, as shown in Figure 12. Two-axis RDA explained 60.81% and 19.18% of the variation, respectively. The angle between the two lines in Figure 12 represents the degree of correlation between the factors and the ordering axis (RDA1 and RDA2, principal components 1 and 2). An acute angle (<90°) indicates a positive correlation, the obtuse angle (>90°) indicates a negative correlation, and the right angle (90°) indicates no correlation. The line length between the arrow and the origin represents the degree of correlation between the factor and the community and species distribution. The longer the line, the greater the correlation. The results showed that the angle between FAAs and *Enterobacter*, *Acinetobacter*, *Pseudomonas*, *Empedobacter*, *Pantoea*, *Comamonas*, and *Chryseobacterium* was small, indicating that there was a strong positive correlation between FAA and these seven genera. The connection between *Lactobacillus* and *Geobacterium* is long; that is, FAAs are highly correlated with *Lactobacillus* and *Geobacterium*, indicating that this bacterial genus is closely related to the production of proteases and enzymes, and proteins are decomposed into FAAs under the action of enzymes. FAAs are formed by enzymes, which break down proteins in large quantities. Therefore, microorganisms in the fermentation system can influence FAA production and metabolism.

### 3.9. Dynamic Changes in BAs in Mao-Tofu

BAs are formed through FAA decarboxylation, which can be induced by microorganisms present in food. The results showed that the existence of microorganisms producing decarboxylase is related to BAs, with the synthesis and activity of decarboxylase being crucial factors in BA production [47,48,49].

In Table 6, seven BAs were detected during Mao-tofu processing, including phenethylamine, putrescine, cadaverine, histamine, tyramine, spermidine, and spermine. However, except for 72 h fermentation, tryptamine was not detected during fermentation. Moreover, the total BA concentration in the fresh Mao-tofu was the lowest, measuring only 20.72 mg/L. In the first four fermentation stages, the BA concentration did not change significantly, but in the middle and late fermentation stages, the BA concentration reached a peak value of 118.39 mg/L at 60 h. Consequently, the development of BA was mainly at 48 h fermentation. These results may relate to the histidine and tyrosine decarboxylase and phenylalanine with certain activity to promote FAA decarboxylation in Mao-tofu to produce BAs.

An important BA associated with food poisoning is tyramine. In this context, poisoning is a “cheese reaction” due to its association with foods containing high tyramine concentrations, primarily found in cheese [50]. Tyramine concentrations <100 mg/kg in this study sample did not pose a potential risk of health problems. The spermine concentration in the fermentation was second only to that of tyramine, and its variation trend closely resembled histamine. Tryptamine, phenylethylamine, and spermidine levels were consistently maintained, emphasizing the relatively low spermidine concentration.

According to the histamine risk assessment conducted by the European Food Safety Authority, up to 100 mg/kg of histamine is not harmful to human health, whereas levels exceeding 200 mg/kg can have toxic effects on individuals [51]. The histamine concentration was the highest among all bioamines produced during Mao-tofu fermentation, and it continued to rise during Mao-tofu processing, reaching a maximum of 74.01 mg/L at 48 h. However, it started to decrease significantly after the processing. In the previous analysis of FAA, the histidine content was the highest at 60 h fermentation. Currently, under the influence of histidine decarboxylase, the histamine content also reached its highest level. Some reports suggested that histamine, the most toxic BA, produces BAs in fermented foods [52]. The concentration fluctuated from 1.00 to 74.01 mg/L, which remained below the acceptable limit of 100 mg/L for fresh matter. These results indicated that the first 60 h fermentation was possibly the peak for producing histidine. From a food security perspective, it is preferable to extend the fermentation process of Mao-tofu beyond this timeframe.

**Figure 13:**
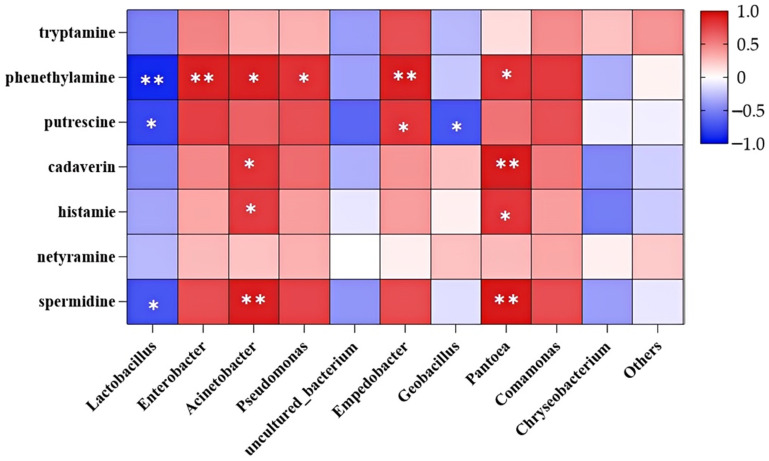
Spearman correlation between dominant bacteria and BAs in Mao-tofu (* indicates a high degree of relevance, ** indicates a higher degree of relevance).

The correlation between bacterial community structures and BAs in Mao-tofu was assessed based on the Spearman correlation test, as shown in Figure 13, which showed the correlation between the top 10 bacterial genera in terms of relative abundance and the seven BAs, with closer to the red colour in the heatmap indicating a stronger positive correlation and closer to the blue colour indicating a higher negative correlation. The results indicated that seven bacterial genera were correlated (*p* < 0.05) with at least one BA.

*Acinetobacter* showed a highly significant positive correlation (*p* < 0.01) with phenethylamine, cadaverine, and histamine, which are common amine-producing genera in fermented foods. *Pseudomonas* showed a significant positive correlation (*p* < 0.01) with phenethylamine and *Pseudomonas*. *Empedobacter* showed a significant positive correlation (*p* < 0.01) with phenethylamine and putrescine. *Pantoea* was significantly positively correlated with phenethylamine, cadaverine, and histamine (*p* < 0.01). *Enterobacter* showed a significant positive correlation with phenethylamine (*p* < 0.05). That is, *Acinetobacter* and *Pantoea* were the main amine-producing microorganisms in the samples of this study. *Lactobacillus* showed a highly significant negative correlation with phenethylamine, putrescine, and spermine (*p* < 0.01), and *Geobacillus* showed a highly significant negative correlation with putrescine (*p* < 0.01). The bacterial genera *Lactobacillus* and *Geobacillus* are commonly found in Mao-tofu, suggesting their potential for degrading BAs. In the previous analysis of bacterial community changes during Mao-tofu fermentation, *Lactobacillus* was the predominant bacteria throughout the entire fermentation process, whereas *Enterobacter*, *Acinetobacter*, and *Pseudomonas* emerged as dominant species at 48 h fermentation. Their abundances reached their peaks at 60 h fermentation. However, there was no significant correlation between the number of BAs and the species of microorganisms in the sample. This proved that the BA formation is independent of microbial species [53]. These results contradicted the findings of Barbieri et al. [54], who reported that *Leuconostoc mesenteroides* could produce histamine, putrescine, and tyramine. They also reported that *Entercoccus faecium* and *Entercoccus faecalis* produced histamine, tyramine, putrescine, and cadaverine. *Leuconostoc* sp. D82 has no BA potential, except for cadaverine, which differed from previous reports. Therefore, it was speculated that BAs are produced due to amino acid decarboxylation by microbial substrate-specific enzymes, and decarboxylase activity is strain dependent rather than genus or species specific. That is, *Lactobacillus* in the Mao-tofu sample in this study may not have the corresponding amine-producing genes and did not result in a significant BA accumulation in the sample. Therefore, further research could isolate *Lactobacillus* from the samples in this study as a potential functional fermenter for inhibiting BA formation.

## 4. Conclusions

In order to investigate the quality formation mechanism of Mao-tofu, the dynamic changes of physicochemical properties, BA concentration, and microbial diversity during the fermentation of Mao-tofu were analysed. The results showed that the fermentation time was positively correlated with the hardness, viscosity, and chewiness elasticity of Mao-tofu, and inversely correlated with the moisture content, pH, and elasticity, and that fermented Mao-tofu was rich in volatiles, with a higher content and a greater variety of organic acids and alcohols. Compared with fresh tofu, the content of proteins and peptides in cooked furikake tofu decreased, while the content of total FAA increased 14-fold. The most abundant amino acids in furikake tofu were Arg, Ala, Lys, Leu, Phe, and Pro, and histamine had the highest concentration of all BAs during fermentation. The relationship between the accumulation of BA and the number of microorganisms was not clear. At the phylum level, there were fungal phyla (*Basidiomycota*, *Ascomycota*, *Mucoromycota*, *Mortierellomycota*, *Glomeromycota*, and *Chytridiomycota*) and two bacterial phyla (Firmicutes and Proteobacteria). At the genus level, there were 10 fungal species (*Trichosporon*, *Apiotrichum*, *Cutaneotrichosporon*, *Candida*, *Diutina*, *Aspergillus*, *Wallemia*, *Debaryomyces*, *Meyerozyma*, and *Cladosporium*). There were also nine bacterial species (*Lactobacillus*, *Enterobacter*, *Acinetobacter*, *Pseudomonas*, *Empedobacter*, *Geobacillus*, *Pantoea*, *Comamonas*, and *Chryseobacterium*). This study aimed to systematically reveal the changes in microbiodiversity and microbiota during Mao-tofu fermentation while also briefly analysing the relationship between FAAs, bioamines, and microbial diversity. The findings provided valuable insights for selecting appropriate microbiota during Mao-tofu fermentation and laid a solid foundation for evaluating the impact of traditional fermented soybean products on human health. Ultimately, this research aimed to develop high-quality fermented soybean products.

## Figures and Tables

**Table 1:** Gradient elution program settings.

Time/min	Velocity of Flow/(mL·min^−1^)	A/%	B/%
0~1.00	0.30	10	90
2.90	0.30	55	45
3.90	0.30	55	45
4.00	0.30	10	90
6.50	0.30	10	90

**Table 2:** Volatile compounds in Mao-tofu samples at different stages of fermentation.

Aroma Compounds	12 h		24 h		36 h		48 h		60 h		72 h	
	Contents (μg/g)	Kinds	Contents (μg/g)	Kinds	Contents (μg/g)	Kinds	Contents (μg/g)	Kinds	Contents (μg/g)	Kinds	Contents (μg/g)	Kinds
Acids	3.389	9	4.402	9	NA	--	NA	--	4.589	9	2.959	6
Esters	3.462	10	2.030	6	NA	--	NA	--	1.617	7	2.222	9
Alcohols	0.934	5	1.085	7	NA	--	NA	--	1.349	7	0.977	6
Aldehydes	1.262	5	0.676	3	NA	--	NA	--	0.366	3	0.270	4
Alkenes	0.704	3	0.512	3	NA	--	NA	--	0.552	3	0.601	3
Ketones	2.90	2	10.73	1	NA	--	NA	--	0.430	1	0.932	2
Phenols	--	--	--	--	NA	--	NA	--	0.272	2	0.150	2
Furans	1.482	1	0.743	1	NA	--	NA	--	0.547	1	0.912	1
Indoles	0.113	1	0.125	1	NA	--	NA	--	--	--	0.105	1
Total	14.246	36	38.733	44.04	NA	--	NA	--	9.722	33	9.128	34

NA, not available; --, not detected.

**Table 3:** Concentrations of different volatile compounds at different fermentation times.

	Aroma Compounds	Contents of Volatile Aroma Compounds in 5 Mao-Tofu Brick Samples/(μg/g)					
		12 h	24 h	36 h	48 h	60 h	72 h
Acids							
1	Acetic acid	1.134	2.308	NA	NA	2.365	1.072
2	Propionic acid	2.134	3.308	NA	NA	3.365	2.072
3	9-decyl acetic acid	--	--	NA	NA	0.11	--
4	Butyric acid	0.079	0.298	NA	NA	0.027	0.012
5	3-methyl butanoic acid	0.134	0.274	NA	NA	0.378	0.212
6	Caproic acid	0.023	0.01	NA	NA	0.017	--
7	Palmitic acid	0.842	0.675	NA	NA	1.034	1.1
8	2-methyl butyric acid	0.257	0.132	NA	NA	0.079	--
9	Oleic acid	0.378	0.356	NA	NA	0.279	0.304
10	Linoleic acid	0.124	0.245	NA	NA	0.3	0.259
Ethyl							
1	Ethyl acetate	1.742	1.207	NA	NA	0.559	1.314
2	Hexyl acetate	0.278	0.214	NA	NA	0.239	0.127
3	Ethyl caproate	0.432	0.319	NA	NA	0.258	0.104
4	Ethyl oenanthate	0.031	--	NA	NA	0.124	0.057
5	Ethyl laurate	0.123	0.111	NA	NA	--	--
6	1-cyclopentene-1-carboxylieacid	0.107	--	NA	NA	--	--
7	Ethyl palmitate	0.147	--	NA	NA	0.145	0.159
8	Ethyl oleate	0.12	--	NA	NA	0.098	0.129
9	Ethyl linoleate	0.293	0.132	NA	NA	0.194	0.235
10	Ethyl 3-phenylpropionate	0.189	0.047	NA	NA	--	0.097
Alcohol							
1	Isoamyl alcohol	0.083	0.124	NA	NA	0.271	0.184
2	1-hexauol	0.345	0.227	NA	NA	0.219	0.327
3	1-octen-3-ol	0.143	0.202	NA	NA	--	--
4	Enanthol	--	--	NA	NA	0.145	0.209
5	Isooctanol	--	0.039	NA	NA	0.122	0.092
6	Octanol	--	0.042	NA	NA	0.095	0.048
7	1-nonamol	0.238	0.264	NA	NA	0.145	0.117
8	Phenylethyl alcohol	0.125	0.187	NA	NA	0.352	--
Aldehydes							
1	(E)-2-heptenal	0.017	0.053	NA	NA	0.044	0.03
2	2-undecenal	0.041	--	NA	NA	--	0.021
3	Nonaldehyde	0.189	--	NA	NA	0.128	0.107
4	Benzaldehyde	0.634	0.381	NA	NA	--	--
5	2-phenyl-2-butenal	0.381	0.242	NA	NA	0.194	0.112
Alkenes							
1	Phenylethylene	0.034	0.142	NA	NA	0.084	0.057
2	Caryophyllene oxide	0.183	0.152	NA	NA	0.173	0.18
3	2-methyl-2-butene	0.487	0.218	NA	NA	0.295	0.364
Ketones							
1	2-octanone	0.237	0.381	NA	NA	0.217	0.251
2	3-octanone	0.124	--	NA	NA	0.213	0.681
Phenols							
1	Guaiacol	--	--	NA	NA	0.178	0.112
2	Phenol4-vinylguaiacol	--	--	NA	NA	0.094	0.038
Furan							
1	2-amyl furan	1.482	0.743	NA	NA	0.574	0.912
Indole							
1	Indole	0.113	0.125	NA	NA	--	0.105

NA, not available; --, not detected.

**Table 4:** Changes in hardness, viscosity, chewiness, and elasticity during the Mao-tofu fermentation process.

Fermentation Time/h	Hardness/%	Viscosity/%	Chewiness/%	Elasticity/%
0	20	9.3	34	100
12	23	11.7	43	93
24	27	13.4	47	87
36	31	15.3	54	81
48	34	19.8	63	78
60	62	27.3	74	73
72	87	43.2	92	69

**Table 5:** Amino acids of Mao-tofu at different fermenting stages.

FAA	Full Names	Amino Acid Content of Huizhou Mao-Tofu Under Different Fermentation Times/mg·kg^−1^						
		0 h	12 h	24 h	36 h	48 h	60 h	72 h
Cys	Cysteine	0.5	1.3	5.2	10.6	16.7	32.5	30.2
Ser	Serine	9.6	14.1	16.3	54.1	125.6	251.9	238.7
Asp	Aspartic Acid	27.8	32.0	40.1	46.1	86.8	159.5	124.7
Gly	Glycine	37.1	50.6	52.5	58.8	66.5	117.2	100.8
Thr	Threonine	7.4	8.0	12.5	25.2	80.2	181.1	174.3
Ala	Alanine	36.7	42.4	55.1	151.8	232.3	524.3	481.4
Glu	Glutamine	33.0	49.3	68.4	95.7	195.8	316.4	285.3
Lys	Lysine	21.0	24.5	30.0	85.9	232.6	504.9	460.2
Pro	Proline	10.4	13.3	19.2	81.2	102.9	333.0	307.6
His	Histidine	29.3	32.3	49.0	69.1	89.9	197.0	167.4
Arg	Arginine	95.6	121.7	150.2	202.4	350.3	546.4	528.2
Val	Valine	2.9	3.5	12.6	41.5	120.2	254.5	224.7
Met	Methionine	0.2	1.0	1.9	9.3	15.7	36.7	34.2
Tyr	Tyrosine	5.4	6.9	9.4	49.5	149.6	250.8	217.5
Ile	Isoleucine	1.7	2.1	5.3	33.5	93.3	189.1	154.0
Leu	Leucine	3.5	4.2	13.3	83.6	206.3	386.9	351.7
Phe	Phenylalanine	6.8	8.1	16.1	80.3	227.1	369.9	344.9
Trp	Tryptophan	0.5	1.5	4.8	9.2	14.3	30.8	29.4
TAA	Total FAA	329	417	562	1188	2406	4683	4255

**Table 6:** BA concentration (mg/kg Mao-tofu) in the examined Mao-tofu and brine.

Mao-Tofu	Amino Acid Content of Huizhou Mao-Tofu Under Different Fermentation Times/mg·L^−1^							
	Tryptamine	Phenethylamine	Putrescine	Cadaverine	Histamine	Tyramine	Spermidine	Total
0 h	2.61	1.76	3.20	--	--	10.05	3.11	20.72
12 h	3.03	1.81	4.38	--	1.00	10.79	0.89	21.90
24 h	2.95	1.89	4.94	--	2.57	10.82	1.04	24.51
36 h	2.97	2.41	3.90	--	26.89	20.90	3.61	60.7
48 h	3.54	6.16	30.42	--	15.47	24.11	1.35	81.05
60 h	2.90	2.41	10.73	--	74.01	26.21	2.13	118.39
72 h	3.10	--	18.43	13.04	9.66	10.19	2.84	57.26

## Data Availability

The original contributions presented in this study are included in the article. Further inquiries can be directed to the corresponding authors.
